# A High‐Throughput Assay for Monitoring and Quantifying Amyloid‐β Accumulation and Clearance in Alzheimer's Disease Cell Models

**DOI:** 10.1111/jnc.70473

**Published:** 2026-05-16

**Authors:** Ajish Ariyath, Fraulein Denise Arigo, Anna Fyfe, W. M. A. D. Binosha Fernando, Ralph Martins, Prashant Bharadwaj

**Affiliations:** ^1^ Centre of Excellence for Alzheimer's Disease Research and Care, School of Medical and Health Sciences, Sarich and Patricia Neuroscience Research Institute Edith Cowan University Joondalup Western Australia Australia; ^2^ Alzheimer's Research Australia Nedlands Western Australia Australia; ^3^ University of Western Australia Medical School Perth Western Australia Australia; ^4^ School of Biomedical Science Macquarie University Sydney New South Wales Australia; ^5^ Curtin Medical School, Curtin Health and Innovation Research Institute (CHIRI), Faculty of Health Sciences Curtin University Western Australia Australia

## Abstract

Amyloidogenic proteins, such as amyloid‐β (Aβ), self‐assemble into cross‐β fibrils whose accumulation is central to Alzheimer's disease (AD). Measuring Aβ aggregation and clearance in living cells remains challenging using current cell‐based assays, which are often low‐throughput or not suited for real‐time monitoring. This study aimed to (1) develop a robust, quantitative, and scalable fluorescence‐based assay using Amytracker to monitor Aβ accumulation and clearance in an Aβ‐producing neuronal cell model, and (2) validate its utility for mechanistic studies and therapeutic screening. We established a plate‐based fluorescence assay using Amytracker in MC65 neuronal AD model expressing the Amyloid precursor protein C‐terminal fragment (APP‐C99) that generates Aβ. Accumulation and clearance of Aβ were quantified by measuring Amytracker fluorescence under basal conditions and after inducing Aβ clearance using a Tet‐suppressible system. We utilized this assay to evaluate cell death inhibitors ferrostatin‐1 and liproxstatin‐1 and proteasome activator IU1. Specificity of the assay for amyloidogenic proteins was assessed by treating wild‐type neuroblastoma cells with Aβ, human islet amyloid polypeptide (hIAPP), or non‐aggregating Aβ controls. Validation included Aβ immunoblotting and cell viability assays. In APP‐C99 expressing cells, elevated Amytracker fluorescence correlated with increased Aβ accumulation and reduced cell viability. Supplementation of ferrostatin‐1, liproxstatin‐1, and IU1, on these cells, markedly reduced Amytracker signal, indicating decreased Aβ burden. Furthermore, the Amytracker assay specifically detected amyloidogenic protein aggregation: wild‐type cells exposed to Aβ42 or hIAPP showed high fluorescence, whereas non‐aggregating Aβ16 peptide did not. The Amytracker assay provides a simple, non‐toxic, and high‐throughput platform for quantifying Aβ accumulation and clearance in live cell models. Its sensitivity, specificity, and compatibility with high‐throughput screening make it a valuable tool for studying Aβ dynamics, interrogating mechanisms of proteostasis, and identifying therapeutic candidates targeting Aβ.

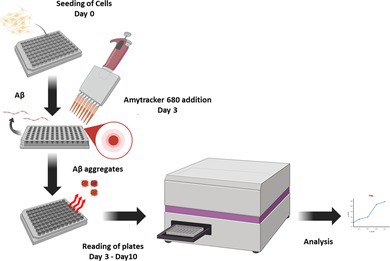

## Graphical abstract

Alzheimer's disease (AD) is primarily caused by the aggregation and clearance of amyloid‐β (Aβ), but there are currently few tools available to track intracellular Aβ dynamics in living cells. Here, we develop a scalable fluorescence assay to identify and measure Aβ aggregation in living neuronal cells using the luminescent conjugated oligothiophene dye Amytracker 680. Amytracker fluorescence tracks Aβ accumulation in the MC65 APP‐C99 model, correlates with toxicity, and decreases when proteostasis pathways are pharmacologically modulated. This live‐cell platform offers a sensitive, high‐throughput method for mechanistic research and therapeutic screening aimed at Aβ aggregation and clearance, as well as real‐time visualization of intracellular amyloid dynamics.

**Abbreviations**
AD: alzheimer's disease
ANOVA: analysis of variance
APP: amyloid precursor protein
APP‐C99: amyloid precursor protein C‐terminal fragment (99 amino acids)
AUC: analytical ultracentrifugation
Aβ: amyloid‐beta
Aβ16: Truncated non‐aggregating N‐terminal fragment of amyloid‐beta
Aβ42: 42‐amino acid isoform of amyloid‐beta
BCA: bicinchoninic acid
BF‐188: benzofuran‐based amyloid imaging probe
CNS: central nervous system
DLS: Dynamic light scattering
DMEM: Dulbecco's Modified Eagle Medium
DMSO: dimethyl sulfoxide
ECL: enhanced chemiluminescence
ELISA: enzyme‐linked immunosorbent assay
FBS: foetal bovine serum
FDDNP: 2‐(1‐{6‐[(2‐Fluoroethyl)(methyl)amino]‐2‐naphthyl}ethylidene)malononitrile
GAPDH: glyceraldehyde‐3‐phosphate dehydrogenase
GFP: green fluorescent protein
HFIP: hexafluoroisopropanol
hIAPP: human islet amyloid polypeptide (amylin)
HP: horseradish peroxidase
LCO: luminescent conjugated oligothiophene
MCI: mild cognitive impairment
MS: mass spectrometry
NIAD‐4: [[5′‐(4‐Hydroxyphenyl)[2,2′‐bithiophen]‐5‐yl]methylene]‐propanedinitrile
PBS: phosphate‐buffered saline
SD: standard deviation
SDS‐PAGE: sodium dodecyl sulfate‐polyacrylamide gel electrophoresis
SEC: size exclusion chromatography
T2D: type 2 diabetes
TBS: tris‐buffered saline
TBS‐T: tris‐buffered saline with Tween‐20
ThT: thioflavin T
USP14: ubiquitin‐specific protease 14

## 1 Introduction

Alzheimer's disease (AD) is widely considered an Aβ clearance‐related disorder in which impaired proteostasis drives progressive amyloid accumulation and neurodegeneration (Cozachenco et al. 2023). Multiple approaches exist for detecting and tracking Aβ accumulation and clearance in AD cell models. Standard immunoassays such as Enzyme Linked Immunosorbent Assay (ELISA) and mass spectrometry (MS) methods remain standard for quantifying Aβ in fluids and tissue homogenates, but these represent endpoint, low‐throughput approaches that are unsuitable for live monitoring in cells. On the other hand, reporter‐based (Gao et al. 2021) and recombinant protein systems, including fusion‐protein constructs and GFP‐tagged Aβ models, have been used to study Aβ accumulation and clearance in high‐throughput formats (Bharadwaj et al. 2010). The APP‐processing and γ‐secretase assays measure Aβ and APP‐derived fragments, providing insight into upstream processing events. However, these models do not exhibit aggregate or oligomer formation, proteostasis dysfunction, or cell death (Riegerová et al. 2021; Mputhia et al. 2019; Zhang et al. 2007). Similarly, studies using exogenous synthetic Aβ peptides in neuronal and non‐neuronal cell lines primarily address cytotoxicity rather than the endogenous Aβ dynamics of Aβ production, aggregation, and clearance (Bharadwaj et al. 2009; Dharmaraj et al. 2022).

Traditional amyloid‐binding dye based assays such as Thioflavin T (ThT) and Congo Red (Biancalana and Koide 2010) are suited for rapid and high‐throughput assays, but have limitations, particularly in cellular assays, due to cytotoxicity and poor membrane permeability (Darghal et al. 2006; Yakupova et al. 2019; Álvarez‐Berbel et al. 2025). ProteoStat is another dye that detects aggregated proteins and can be used in both fixed and live cells; however, it requires cell‐permeabilization steps for optimal staining (Shen et al. 2011). Congo Red derivatives such as X‐34 are fluorescent, blood–brain‐barrier‐permeable probes used for visualizing amyloid plaques in vivo. Other amyloid‐binding ligands such as NIAD‐4, BF‐188, curcumin, and radioactive probes including FDDNP have been developed for experimental imaging of amyloid pathology in both animal models and clinical studies; however, these dyes have shown low specificity, limited live‐cell applicability, and cytotoxic effects (Álvarez‐Berbel et al. 2025; Owyong et al. 2025). Collectively, these limitations highlight the critical need for physiologically relevant human‐derived cell models with high throughput capacity and the capability to monitor Aβ accumulation and clearance and exhibit Aβ‐mediated toxicity (Bharadwaj et al. 2009; Dharmaraj et al. 2022).

In this study, we utilized the human CNS‐derived neuroblastoma cell line (MC65), which stably expresses the APP‐C99 fragment under a tetracycline (Tet) suppressible promoter and produces Aβ via γ‐secretase cleavage. The MC65 cells also exhibit key features of AD‐related proteostasis dysfunction, including impaired autophagy and proteasome activity, closely resembling pathologies observed in post‐mortem AD brain tissue (Mputhia et al. 2019; Bharadwaj and Martins 2020). The main objective of this study was to establish a robust and scalable assay for monitoring Aβ aggregation and clearance in live neuronal cells. To this end, we developed a plate‐based high‐throughput quantitative assay utilizing the fluorescent probe Amytracker, a luminescent conjugated oligothiophenes (LCOs) in the APP‐C99/Aβ‐producing MC65 neuronal model. The Amytracker assay was evaluated for its ability to accurately measure Aβ accumulation and clearance using the established Tet‐suppressible system (Mputhia et al. 2019). The assay was subsequently employed to investigate inhibitors of Aβ‐mediated cell death and proteasome enhancers that are known to promote aggregate protein clearance, such as tau and α‐synuclein. Furthermore, assay specificity for amyloidogenic proteins was validated by comparing responses in wild‐type SH‐SY5Y neuroblastoma cells treated with Aβ42 or human islet amyloid polypeptide (hIAPP) versus a non‐aggregating Aβ16 peptide. Collectively, this work establishes the Amytracker assay as a non‐toxic, quantitative, and high‐throughput platform suitable for real‐time assessment of Aβ aggregation and clearance, with broad applicability for mechanistic and therapeutic discovery studies in AD research.

## 2 Materials and Methods

### 2.1 Maintenance and Culturing of MC65 and SH‐SY5Y Cells

MC65 human neuroblastoma cells (RRID: CVCL_5712) were cultured in DMEM/F12 (Thermo Scientific, 11320082) supplemented with 10% foetal bovine serum (FBS), (Thermo Scientific, 10100147), 1 μg/mL tetracycline (Merck, T7660) with 0.2 mg/mL G418 (Merck, G5013) for routine maintenance. 0.4 mg/mL G418 and 1 μg/mL tetracycline were used at the time of selection, and kept at 37°C in a humidified atmosphere containing 5% CO_2_. To suppress Aβ production, cells were maintained in medium containing 1 μg/mL tetracycline (+Tet; Sigma) (Sopher et al. 1994). Aβ expression was induced by switching to tetracycline‐free medium (‐Tet). Cells were stored in liquid nitrogen in 10% dimethylsulfoxide (DMSO, Merck, D8418) in FBS containing 0.1 μg/mL tetracycline. For experiments, G418 was completely removed, and cells were plated in DMEM/F12 with 10% FBS with or without tetracycline. After 3 days, the media was replaced with opti‐MEM (Thermo Scientific, 22600134), followed by treatments. SH‐SY5Y human neuroblastoma cells (RRID: CVCL_0019; ATCC CRL‐2266) were cultured in Dulbecco's Modified Eagle Medium (DMEM; Gibco, 10564029) enriched with 10% FBS. Cells were plated at equal densities in all wells to ensure that the starting cell numbers were comparable.

### 2.2 Cell Line Authentication and Quality Control

Cells were maintained and authenticated according to American Type Culture Collection guidelines include passage number control, standardized storage and culture conditions, and regular morphology and growth monitoring. Functional validation of a genetically modified cell line expressing Aβ was conducted every 3–6 months, assessing cell viability, Aβ production, and response to control compounds. External laboratories independently validated the model (not published data), and key findings were cross‐checked against published data, including responses to ferroptosis inhibitors, to ensure reproducibility and reliability (Huang et al. 2020). The cell lines are not listed as commonly misidentified by the International Cell Line Authentication Committee. The maximum passage numbers used in this study were P15 for MC65 cells and P14 for SH‐SY5Y cells.

### 2.3 Pharmacological Compound Preparation

Pharmacological compounds IU1 (2.5 μM; Sigma, I1911), a USP14 inhibitor known to enhance proteasomal degradation (Lee et al. 2010); liproxstatin‐1 (500 nM; Sigma, SML1414); and ferrostatin‐1 (500 nM; Sigma, SML0583), both inhibitors of ferroptosis and lipid peroxidation implicated in neurodegenerative disease models, were used in this study (Friedmann Angeli et al. 2014). All compounds were dissolved in DMSO and diluted in culture medium immediately prior to use.

### 2.4 Fluorescent Staining and Quantification With Amytracker 680

Live‐cell detection of intracellular Aβ aggregates was carried out using Amytracker 680 (Ebba Biotech, A680‐A‐100), a luminescent conjugated oligothiophene dye known for its high specificity toward β‐sheet‐rich amyloid structures (Aslund et al. 2009). MC65 cells were cultured in DMEM/F12 supplemented with 10% FBS in the presence or absence of tetracycline and the respective treatment compounds. After 3 days, the medium was replaced with Opti‐MEM, and cells were washed twice with fresh Opti‐MEM. Amytracker 680 (1 mg/mL) was then added to the culture media in 1/500 dilution (2 μg/mL final concentration) and incubated for 30 min at 37°C in a humidified 5% CO_2_ incubator. Following incubation, cells were washed three times with Opti‐MEM and replenished with fresh Opti‐MEM with or without the treatment compounds. Fluorescence was quantified daily up to 10 days, with excitation/emission wavelengths set to 530/680 nm on the Vantastar microplate reader (BMG Labtech). On Day 6, cells were imaged for qualitative analysis of Aβ accumulation using a Nikon Eclipse Ti2 fluorescence microscope. For the Amytracker assay, we employed a greater seeding density of 0.9 × 10^5^ cells/100 μL/well in a 96‐well plate (Corning, CLS4580) in contrast to the viability assay, which utilized 0.5 × 10^5^ cells/100 μL/well. The cell viability experiment lasts for 6 days, while the Amytracker assay extends for 10 days; therefore, a higher density was required to delay cell death and maintain cultures until the 10th day. Quantification of fluorescence intensity was performed using ImageJ software, and statistical analysis was conducted in GraphPad Prism 8.0.2 (Ariyath et al. 2022).

### 2.5 Tet‐Spiking Assay

The clearance of APP‐C99/Aβ was evaluated in MC65 cells, a human‐derived cell line that generates Aβ via the inducible expression of the amyloid precursor protein fragment C99. MC65 cells were cultured without tetracycline for 3 days to induce APP‐C99/Aβ production, then treated with tetracycline (1 μg/mL) on Day 5 to inhibit APP‐C99/Aβ production. Cell lysates were collected on Day 5 (before spike) and Day 6 (the day after spike) to measure levels of Aβ by western immunoblotting analysis. To assess intracellular aggregate clearance in the Amytracker assay, tetracycline (1 μg/mL) was reintroduced on Day 5 (Tet‐spiking), and cells were monitored for Aβ clearance till Day 9.

### 2.6 Western Blot and Protein Analysis

Aβ levels were quantified by analyzing proteins through denaturing sodium dodecyl sulfate‐polyacrylamide gel electrophoresis (SDS‐PAGE) followed by western immunoblotting, as previously outlined (Bharadwaj et al. 2012b). Cells were washed with ice‐cold Phosphate buffer saline (PBS; Merck, 11 666 789 001), and lysed in a Triton buffer (0.7% SDS, 0.3% Triton X‐100, 1X PBS) containing 1X protease and phosphatase inhibitors (Roche) for intracellular protein extraction. Lysates were subjected to centrifugation at 18000*g* for 10 min at 4°C, and the supernatants were harvested for protein analysis. Protein concentrations were quantified using the micro bicinchoninic acid (BCA) protein assay kit (Thermo Fisher Scientific, 23 235). Equal quantities of protein (20–30 μg) were separated on 4%–12% Bis‐Tris gels or 4%–12% Bolt Gels (Thermo Scientific, NW04125BOX). The proteins were transferred to nitrocellulose membranes utilizing the Bio‐Rad semi‐dry transfer system (Bio‐Rad, 1 704 270). Membranes were incubated in 5% skim dry milk in TBS‐T (0.1% Tween‐20) for 1 h at room temperature, pH 7.4. Primary antibodies Aβ (6E10, Covance, SIG‐39300, 1:1000, RRID: AB_2564652) and GAPDH (Cell Signaling Technology, 5174, 1:1000, RRID: AB_561053) were diluted in TBST [TBS containing 0.5% skim milk and 0.05% Tween‐20 (Sigma, P7949)]. Incubation was conducted overnight at 4°C, followed by three 5‐min washes in TBST and subsequently in TBS. Horseradish peroxidase (HP) conjugated secondary antibodies, anti‐mouse (GE Healthcare, GENA931, 1:5000, RRID:AB_772210) and anti‐rabbit (GE Healthcare, GENA934, 1:5000, RRID:AB_772206), were diluted in 0.5% skim milk in TBST at a concentration of 1:5000 and incubated with the membranes for 1 h. Following three washes with TBST and TBS for 5 min each, the membranes were incubated for 2 min with HP reactive ECL (Enhanced Chemiluminescence) reagent (Merck, GERPN2106). The membranes were visualized utilizing a Vilber Fusion FX6 imager. The immunoreactive bands were subsequently quantified utilizing Biorad Image Lab software (version 6.0). Quantitative western blotting data were used to validate Amytracker 680 fluorescence as a measure of intracellular Aβ aggregates in live‐cell imaging.

### 2.7 Peptide Preparation and Treatment

Synthetic peptides, including Aβ42 (AS‐20276), Aβ16 (AS‐62897), and human islet amyloid polypeptide (hIAPP; AS‐60254), and novel peptides, were prepared as previously described (Dharmaraj et al. 2022). Briefly, lyophilized peptides were dissolved in hexafluoroisopropanol (HFIP; Merck, 105 528) to monomerize aggregates, aliquoted, and evaporated under a gentle nitrogen stream to form peptide films. Films were stored at −20°C until use. Prior to cell treatment, films were reconstituted in dimethyl sulfoxide (DMSO) and diluted in cell culture media to the desired concentrations. SH‐SY5Y cells were incubated for 24 h with peptides in 5, 15, and 30 μM concentrations, followed by staining with Amytracker 680 for 30 min at 37°C in a humidified 5% CO_2_ incubator to evaluate amyloid formation and dye specificity (Aslund et al. 2009).

### 2.8 Statistical Analysis

Statistical analyses were carried out using GraphPad Prism 8.0.2. The sample size for each experiment was not calculated by a formal calculation; rather, the settings were based on prior similar experimental designs (Ariyath, Hone, et al. 2026; Ariyath, Arigo, et al. 2026; Bharadwaj et al. 2020; Ruankham et al. 2026). There were no outlier experiments performed; all biological replicates (*n* = 4 independent experiments) were included in the statistical evaluation. The data is reported as column scatter plot, where the bars represent the mean ± SD. Individual data points from *n* = 4 independent experiments are shown as overlaid symbols. The normality of the data distribution was determined using the Shapiro–Wilk test in GraphPad Prism. Parametric statistical tests were performed since analysis of variance (ANOVA) is robust to moderate deviations from normality, and the group sizes were equal. When a single independent variable was analyzed, one‐way ANOVA was used; when experiments involved two independent variables, two‐way ANOVA was used to evaluate both main effects and their interaction. Tukey's test was used for comparisons between all groups, Dunnett's test when treatments were compared with a single control, and Sidak's test for pairwise comparisons after two‐way ANOVA. Values from cell viability experiments were adjusted to the relevant control and reported as a percentage of control. *p*‐values < 0.05 indicated statistical significance. In longer fluorescence time‐course experiments (e.g., 9‐day monitoring), the same wells were tracked repeatedly over multiple days within each experiment. These studies were independently repeated four times, and the results are presented as descriptive line graphs to demonstrate temporal trends in fluorescence rather than for statistical comparison.

## 3 Results

### 3.1 Monitoring Aβ Accumulation and Toxicity in an APP‐C99/Aβ‐Producing AD Cell Model

Current methods for studying protein aggregation include biophysical techniques and fluorescence‐based probes. Many approaches, however, require purified proteins or are limited by cytotoxicity, poor specificity, spectral interference, and suitability for live‐cell imaging or high‐throughput screening. As a result, improved tools are required to reliably monitor amyloid aggregation and clearance in cellular systems (Álvarez‐Berbel et al. 2025; Chaudhuri et al. 2014; Roberti et al. 2007; Styren et al. 2000; Ono et al. 2011; Velander et al. 2017; Shin et al. 2011).

Recently, luminescent conjugated oligothiophenes (LCOs) have emerged as highly specific and versatile dyes. In contrast to conventional probes, LCOs possess conformationally sensitive fluorescence spectra that allow for the distinction between amyloid strains and polymorphic aggregates. Nonetheless, their validation in live human cell systems remains constrained (Klingstedt et al. 2013; Nyström et al. 2017; Urbanek et al. 2025). In this study, we used Amytracker 680 to develop a plate‐based high‐throughput screening assay in an AD neuronal cell model to monitor Aβ aggregation and clearance. For this purpose, we used a human central nervous system (CNS) derived cell line, MC65, which generates Aβ by γ‐secretase cleavage from a stably transfected C99 fragment of the APP on a tetracycline promoter (‐Tet) system to model AD. MC65 is a widely used model, and our recent study has demonstrated that this model recapitulates proteostasis dysfunction similar to AD postmortem brains (Mputhia et al. 2019; Bharadwaj and Martins 2020; Bharadwaj et al. 2012a).

**FIGURE 1:**
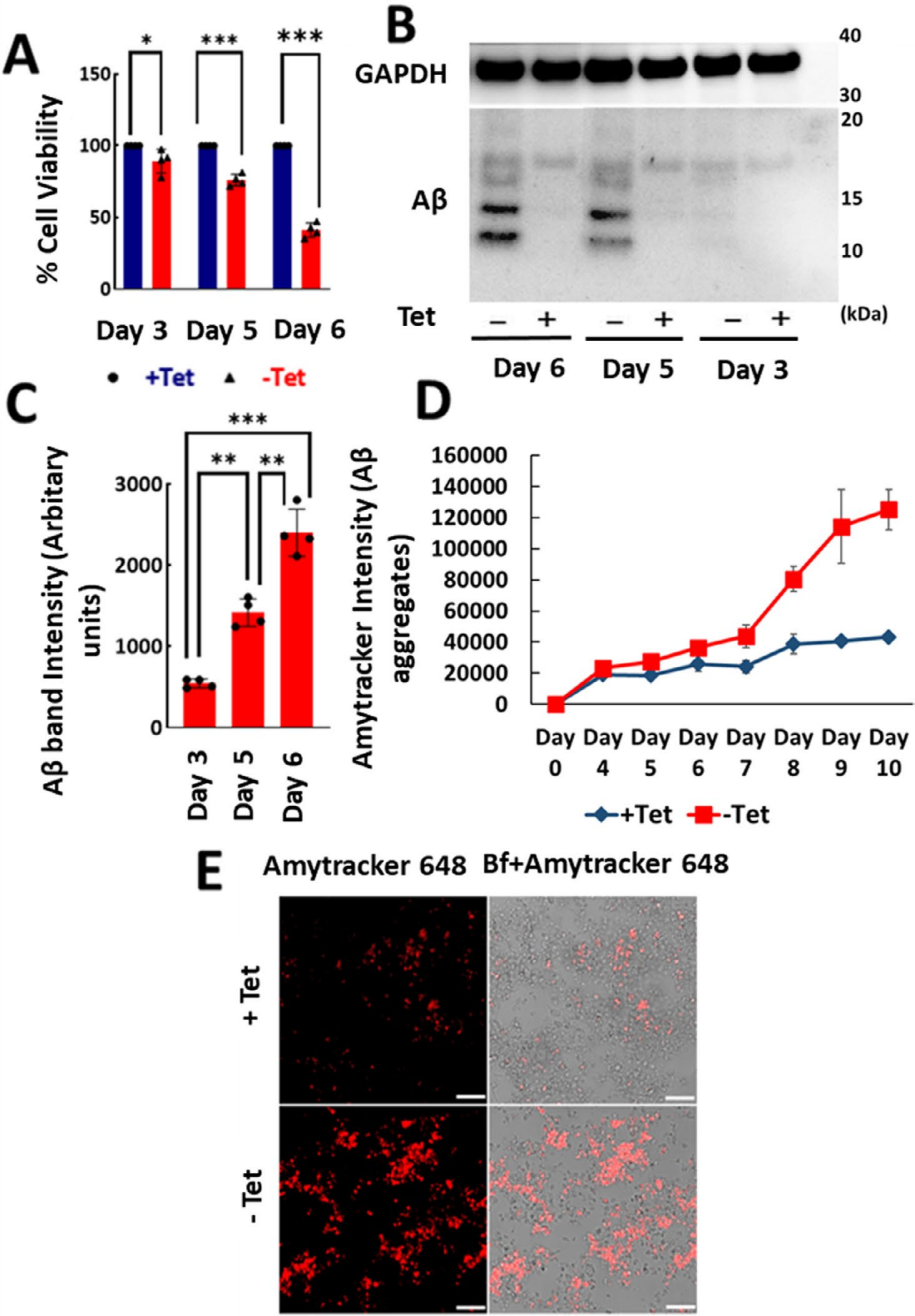
Aβ aggregate formation in APP‐C99/Aβ‐producing and non‐producing MC65 cells. (A) MTS assay showing cell viability in MC65 cells cultured without tetracycline (‐Tet; APP‐C99/Aβ‐producing) or with tetracycline (+Tet; non‐producing) over 3, 5, and 6 days. Viability decreased by ~10% on Day 3 (*p = 0.0075), ~20% on Day 5 (***p < 0.0001), and ~60% on Day 6 (***p < 0.0001). The data was normalized to the +Tet control condition (B) Western blot analysis confirming Aβ aggregate accumulation corresponding to cell viability changes. (C) Quantification indicated significant increase in Aβ accumulation between Day 3 and 5 (**p = 0.0004), Day 3 and 6 (***p < 0.0001), and Day 5 and 6 (**p = 0.0002). (D) Quantification of Aβ aggregates using Amytracker dye over 10 days in MC65 cells. Aβ levels increased from Day 4 and peaked by Day 10 in ‐Tet cells, whereas +Tet cells remained stable. (E) Representative fluorescence images showing Aβ accumulation in ‐Tet and +Tet conditions. ‐Tet cells exhibit higher Aβ aggregates, consistent with previous data (A–D). In scatter plot graphs, each dot represents a single data point. Quantified data in scatter plots and line graphs are reported as mean ± SD from *n* = 4 different experiments. Scale bar: 10 μM.

We first assessed whether Aβ accumulation in the MC65 model correlates with cytotoxicity. Cell viability was assessed using the MTS (soluble tetrazolium) assay on days 3, 5, 6 in MC65 cells. A two‐way ANOVA following normal distribution revealed significant effects of treatment (*F* (1,18) = 316.5, *p* < 0.0001) and time (*F* (2,18) = 67.73, *p* < 0.0001), as well as a significant treatment × time interaction (*F* (2,18) = 67.73, *p* < 0.0001). Sidak's multiple comparisons test revealed a significant decline in viability in cells cultured without Tet (‐Tet), which induces APP‐C99/Aβ production in comparison to +Tet healthy cells. Viability decreased by ~10% on Day 3 (*p = 0.0075), ~20% on Day 5 (***p < 0.0001), and ~60% on Day 6 (***p < 0.0001). The data was normalized to the +Tet control condition (Figure 1A). Aβ immunoblot analysis corroborated with the cell viability data. Aβ was mostly undetectable on Day 3 after APP‐C99 induction, with a progressive increase in its accumulation by Day 5 (48 h post‐induction) and Day 6 (72 h). Because cell death peaked at Day 6, Aβ immunoblot analyses were performed only up to that time point (Figure 1A–C). Western blot results showed Aβ accumulation grew gradually over time (Figure 1B). After quantification a one‐way ANOVA following normal distribution found a significant effect of time (*F* (2,9) = 89.30, *p* < 0.0001) in the ‐Tet group. Tukey's multiple comparisons test revealed significant increase in Aβ accumulation between Day 3 and 5 (**p = 0.0004), Day 3 and Day 6 (***p < 0.0001), and Day 5 and Day 6 (**p = 0.0002). In comparison to Day 3, the signal increased by ~2.6‐fold at Day 5 and ~4.4‐fold at Day 6, indicating a significant time‐dependent increase in Aβ accumulation (Figure 1C). The data overall confirmed that higher levels of Aβ accumulation were associated with increased toxicity and cell death.

To visualize Aβ accumulation in cells, we treated APP‐C99/Aβ‐producing cells (‐Tet) and control cells (+Tet) with Amytracker 680, followed by quantification of fluorescence (Ex‐530 nm/Em‐680 nm) using a fluorescence plate reader (VANTAstar, BMG Tech). Quantitative fluorescence analysis revealed a time‐dependent increase in Amytracker signal from day 4 (24 h post induction), with a peak around day 9 in ‐Tet cells, whereas +Tet cells maintained baseline fluorescence levels. The Amytracker signal showed a similar trend with both Aβ levels (by western blot) and cytotoxicity (by MTS assay) (Figure 1D). Live fluorescence microscopy performed on day 6 further demonstrated robust intracellular Aβ aggregation in Aβ producing cells (‐Tet), with intense punctate cytoplasmic fluorescence. Wild type control cells (+Tet) showed a minimal or baseline fluorescence similar to Figure 1D (Figure 1E). Untreated cells without Amytracker dye were used as controls to account for autofluorescence. These findings demonstrated the use of Amytracker 680 as a sensitive, live‐cell tool for detecting and quantifying Aβ accumulation in the MC65 AD cell model. We further evaluated the Amytracker assay in quantifying Aβ clearance using our previously reported tet spiking assay.

### 3.2 Evaluating Aβ Clearance in AD Cell Model Using Tet‐Spiking Assay

Impaired clearance of Aβ is considered to be the main causative factor in sporadic AD (Kamatham et al. 2024; Liu, Zhang, and Wang 2024) and a large body of evidence, including clinical studies involving cerebrospinal fluid Aβ turnover analyses have revealed a significant decline in Aβ clearance in sporadic AD patients (Bateman et al. 2012; Mawuenyega et al. 2010; Cheng et al. 2020; Liu, Haziyihan, et al. 2024). The brain employs multiple mechanisms for Aβ clearance, which include both enzymatic and non‐enzymatic pathways, which can be mediated via both extracellular and intracellular pathways.

**FIGURE 2:**
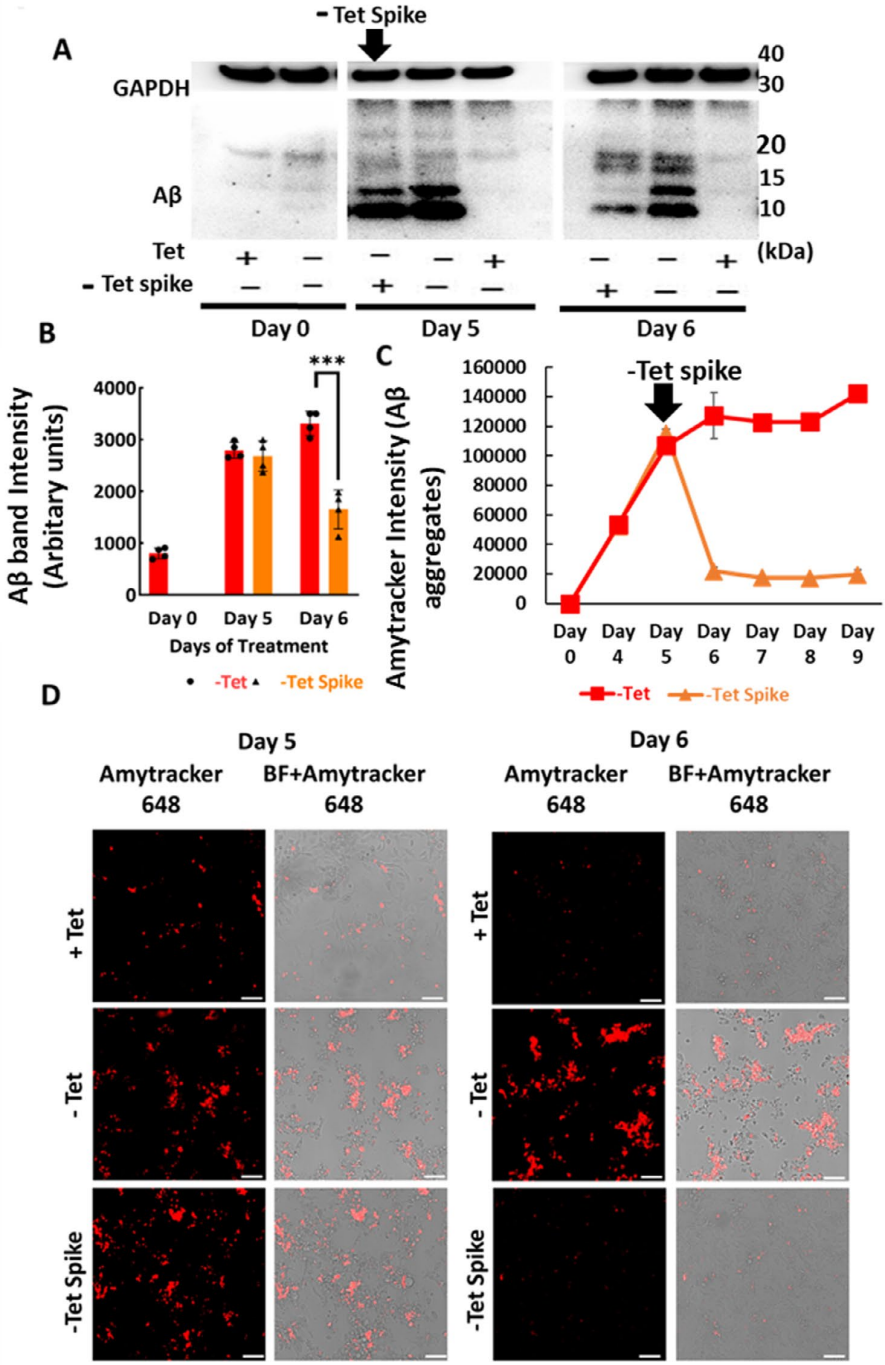
Tetracycline spiking assay to assess Aβ aggregate clearance in APP‐C99/Aβ‐producing MC65 cells. MC65 cells were cultured without tetracycline (‐Tet) for 5 days to induce APP‐C99/Aβ production, followed by tetracycline spiking on day 5 to suppress further expression. (A) Representative western blot image showing Aβ levels in cell lysates collected at Day 0, day 5, and day 6. (B) Quantification of Aβ levels in ‐Tet and ‐Tet Spike groups. The ‐Tet Spike group's Aβ protein levels were significantly reduced at Day 6 (***p < 0.0001) in comparison to the ‐Tet group. (C) To further validate Aβ‐specific binding and clearance, Amytracker dye was used to assess Aβ aggregate accumulation post‐spiking. MC65 cells were cultured without tetracycline (‐Tet) for 5 days to induce APP‐C99/Aβ production, followed by tetracycline spiking on day 5 to suppress further expression. Post‐spiking, a significant reduction in Aβ aggregates was observed in the ‐Tet Spike group from day 5 onward, whereas ‐Tet cells continued to show progressive accumulation up to day 9. (D) Cells were stained with Amytracker dye on Days 5 and 6. Consistent with previous findings, Tet spiking reduced punctate fluorescence signals. By Day 6, Tet‐spiked cells showed fluorescence patterns comparable to those of +Tet control cells. In scatter plot graphs, each dot represents a single data point. Quantified data in scatter plots and line graphs are reported as mean ± SD from *n* = 4 different experiments. Scale bar: 10 μM.

To assess the utility of Amytracker 680 for monitoring the clearance of Aβ, we adapted the tet spiking assay using the APP‐C99/Aβ cell model (Mputhia et al. 2019). Aβ levels were quantified over time using both immunoblotting and the Amytracker assay. Cells were cultured under ‐Tet conditions for 5 days (post induction) to induce APP‐C99 production and allow Aβ accumulation, followed by the reintroduction of tetracycline (‐Tet Spike) on Day 5 to halt further production. This experimental setup enabled us to monitor Aβ clearance in a controlled, time‐resolved manner. Aβ immunoblotting analysis of cell lysates collected at Day 0, 5, and 6 with and without tetracycline spiking revealed a marked decline in Aβ protein levels in the ‐Tet Spike group (Figure 2A). Furthermore, Aβ immunoblot analysis showed a significant reduction in Aβ levels (Figure 2B). Two‐way ANOVA following normal distribution showed significant effects of time (*F* (2,18) = 162.3, *p* < 0.0001) and treatment (*F* (1,18) = 38.57, p < 0.0001), as well as a significant time × treatment interaction (*F* (2,18) = 31.62, *p* < 0.0001) on Aβ protein levels. Sidak's multiple comparisons test found no significant difference between ‐Tet and ‐Tet Spike Aβ protein levels on Day 0 and Day 5. However, Aβ protein levels were significantly reduced in the ‐Tet Spike condition at Day 6 (***p < 0.0001), with ‐Tet cells having ~2‐fold higher Aβ levels than ‐Tet Spike cells (Figure 2B). On the other hand, cells kept in continuous ‐Tet conditions showed sustained or elevated Aβ levels, suggesting continuous accumulation of Aβ. The Amytracker assay showed a marked reduction in fluorescence in the ‐Tet Spike group starting on Day 5 and continuing through Day 9, which was consistent with the immunoblotting data. Conversely, the ‐Tet group exhibited progressive accumulation of Aβ aggregates over the same period. Next, we stained cells with Amytracker dye on Days 5 and 6. Consistent with previous findings, punctate staining patterns were significantly reduced in cells both before and after Tet spiking. Tet‐spiked cells on Day 6 closely resembled +Tet control cells. Together, these results demonstrate that the Amytracker assay is highly sensitive and reliably detects changes in Aβ levels, clearly aligning with the Aβ immunoblotting data. We further evaluated if the Amytracker assay can be adapted to test drug candidates that target Aβ clearance.

### 3.3 Assessing the Effect of Aβ Targeting Drugs Using the Amytracker Assay in the AD Cell Model

**FIGURE 3:**
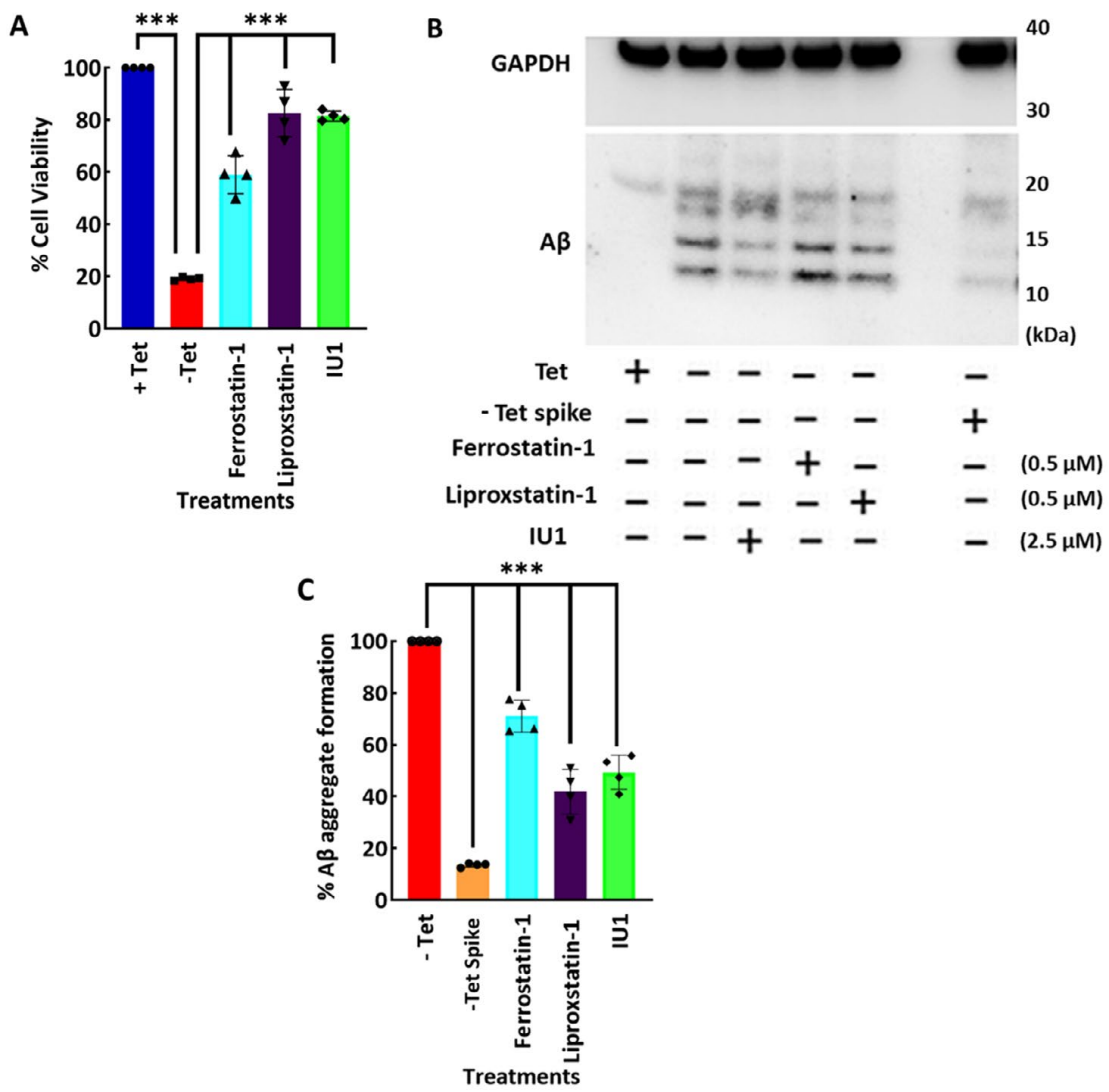
Liproxstatin‐1, Ferrostatin‐1, and IU1 reduce Aβ aggregate formation in APP‐C99/Aβ‐producing MC65 cells. (A) MTS assay showing cell viability in MC65 cells cultured with (+Tet; non‐producing) or without tetracycline (‐Tet; APP‐C99/Aβ‐producing) on Day 6. Cells were cultured without tetracycline for 3 days to induce APP‐C99/Aβ expression, followed by treatment with Liproxstatin‐1, Ferrostatin‐1 (ferroptosis inhibitors), or IU1 (USP14 inhibitor). By Day 6, ‐Tet cells had a significantly lower viability of ~20% compared to the +Tet controls. Ferrostatin‐1 treatment increased cell viability to ~60% (***p < 0.0001). Similarly, Liproxstatin‐1 and IU1 restored viability to ~83% and ~81% (both ***p < 0.0001), respectively. (B) Western blot analysis of Aβ accumulation showing increased levels in ‐Tet cells from Day 4, peaking at Day 6, consistent with the reduced viability observed. (C) Quantification of western blot analysis showed a significant reduction in Aβ levels in a similar pattern to Figure 3A. Treatment with Ferrostatin‐1, Liproxstatin‐1, and IU1 brought Aβ levels to ~70%, ~40%, and ~50% (all ***p < 0.0001) compared to ‐Tet. In the scatter plot graphs, each dot represents a single data point. Quantified data in scatter plots graphs are reported as mean ± SD from *n* = 4 different experiments.

To evaluate the utility of the Amytracker assay in drug screening, we treated the APP‐C99 producing AD cell model with known modulators of Aβ toxicity followed by quantification of Aβ levels using the Amytracker assay and immunoblotting analysis. We also concurrently investigated cell viability and Aβ levels in cells using fluorescence microscopic imaging. Specifically, we assessed the effects of liproxstatin‐1 and ferrostatin‐1, two ferroptosis inhibitors that have been shown to protect MC65 cells (Huang et al. 2020; Skouta et al. 2014). Additionally, we tested a novel small molecule, IU1, an inhibitor of the deubiquitinating enzyme USP14, which has been implicated in proteostasis regulation and clearance of aggregate proteins (Lee et al. 2010). Treatment with Liproxstatin‐1, Ferrostatin‐1, and IU1 significantly improved the viability of the APP‐C99/Aβ producing cells (‐Tet). A one‐way ANOVA following normal distribution revealed a significant effect of treatment on cell viability (*F* (4,15) = 138.7, *p* < 0.0001). Similar to our previous findings, ‐Tet cells had a significantly lower viability of ~20% compared to the +Tet controls. Ferrostatin‐1 treatment increased cell viability to ~60% (***p < 0.0001), a ~3‐fold increase compared to ‐Tet cells. Similarly, Liproxstatin‐1 and IU1 restored viability to ~83% and ~81% (both ***p < 0.0001), respectively, resulting in a ~4‐fold increase in viability compared to ‐Tet (Figure 3A). In addition, immunoblotting analysis of the cells treated with Liproxstatin‐1, Ferrostatin‐1, and IU1 showed a decrease in total Aβ levels (Figure 3B). A one‐way ANOVA following normal distribution showed a significant effect of treatment (*F* (4,15) = 132.6, *p* < 0.0001). According to Dunnett's multiple comparisons test, treatment groups significantly reduced Aβ levels (all groups ***p < 0.0001). Treatment with Ferrostatin‐1, Liproxstatin‐1, and IU1 brought Aβ levels to ~70%, ~40%, and ~50% (***p < 0.0001) compared to ‐Tet (Figure 3C). Similar to the previous result in Figure 2, Tet Spike also reduced Aβ levels significantly. Notably, the decrease in Aβ levels in the various treatment groups closely correlated with the observed increase in cell viability (Figure 3A–C), demonstrating a direct link between Aβ burden and cytotoxicity in this cell model.

**FIGURE 4:**
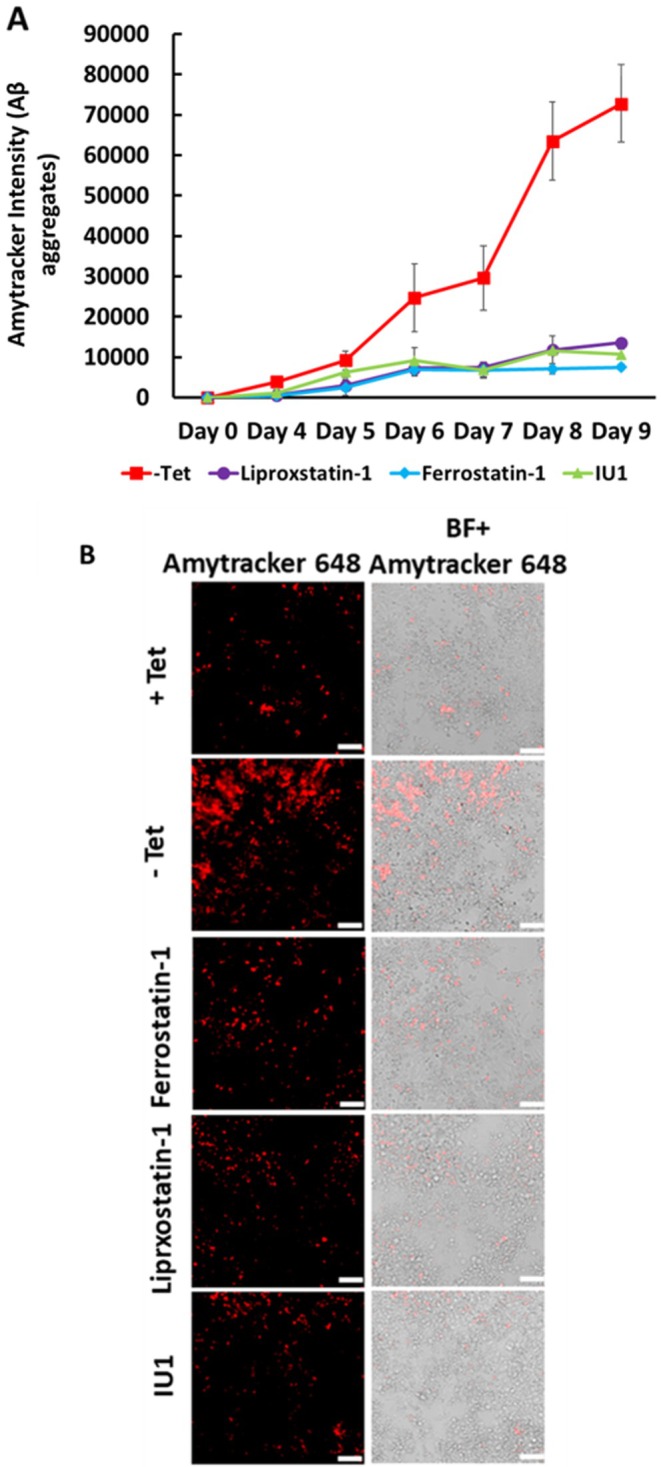
Quantitative and imaging analysis of Aβ aggregate accumulation in MC65 cells. (A) Quantification of Aβ aggregates using Amytracker dye over a 9‐day period. In untreated‐Tet cells, Aβ levels progressively increased, peaking at day 9, Treatment with Liproxstatin‐1, Ferrostatin‐1 and IU1 reduced Aβ accumulation, whereas all treatments effectively suppressed accumulation compared to untreated ‐Tet cells. (B) Representative immunofluorescence images showing Aβ aggregates in MC65 cells. ‐Tet cells exhibited pronounced Aβ accumulation, consistent with quantitative results. Quantified data in line graphs are reported as mean ± SD from *n* = 4 different experiments. Scale bar: 10 μM.

Next, we used the Amytracker assay in the AD cell model to assess the effects of various Aβ‐targeting drugs. Quantitative fluorescence analysis showed a time‐dependent increase in Amytracker fluorescence in ‐Tet cells starting around Day 4 and peaking around Day 9 (Figure 4A), which is similar to the pattern seen in Figure 1. Untreated ‐Tet cells showed a steady and progressive rise in Amytracker fluorescence from Days 4 to 9, indicating the continuing build‐up of Aβ (Figure 4A). Amytracker fluorescence was lower in cells treated with Liproxstatin‐1, Ferrostatin‐1, or IU1 than in untreated ‐Tet cells, which is in line with the findings in Figure 3A,B.

Immunofluorescence imaging provided further qualitative validation of the above findings. In ‐Tet cells, widespread intracellular Aβ aggregation, characterized by intense, punctate staining patterns consistent with the presence of β‐sheet‐rich aggregates, was observed (Figure 4B). This pronounced accumulation corresponded with the elevated Aβ levels detected by immunoblotting and the decreased cell viability measured by MTS assay (Figure 3A,B). In contrast, cells treated with Liproxstatin‐1, Ferrostatin‐1, and IU1 exhibited a reduction in punctate staining patterns resembling the +Tet control cells (Figure 4B).

### 3.4 Detection of Amyloidogenic and Non‐Amyloidogenic Proteins Using the Amytracker Assay

Building on our findings from Figures 1, 2, 3, 4, we next investigated whether the Amytracker assay is specific to amyloid protein accumulation (Dharmaraj et al. 2022). For this experiment, we treated wild type neuroblastoma cells (SH‐SY5Y) with amyloidogenic peptides Aβ42 or human islet amyloid polypeptide (hIAPP or amylin), a hormone co‐secreted with insulin by pancreatic β‐cells. Similar to Aβ's pathogenic role in the brain, amylin aggregation is strongly associated with β‐cell dysfunction in type 2 diabetes (T2D) pancreas, and is also implicated in the increased risk of dementia in T2D patients (Dharmaraj et al. 2022; Lee et al. 2010; Bharadwaj et al. 2020; Bharadwaj et al. 2017). Aβ16, a truncated non‐aggregating N‐terminal fragment of Aβ, was used as a non‐amyloidogenic protein control.

**FIGURE 5:**
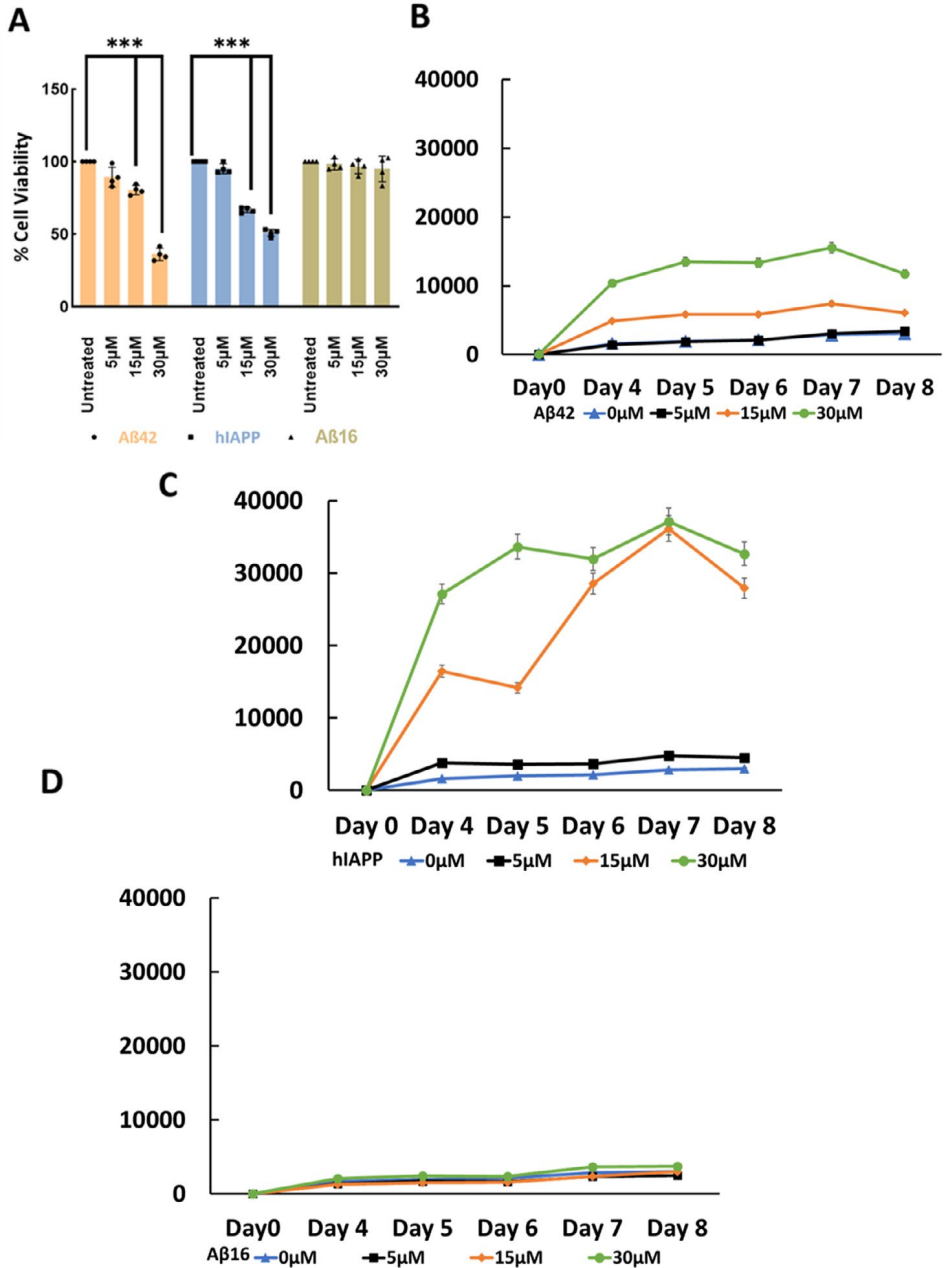
Detection of amyloidogenic and non‐amyloidogenic proteins using the Amytracker assay. (A) Viability and amytracker fluorescence analysis of wild type SH‐SY5Y cells treated with increasing concentrations of Aβ42, hIAPP, and Aβ16 peptides (5, 15, and 30 μM) of synthetic. Aβ42 decreased cell viability by 9%, 20% (***p < 0.0001), and 65% (***p < 0.0001) at doses of 5, 15, and 30 μM, respectively. In contrast, hIAPP caused a minor drop at 5 μM (~5%, not significant), but significantly decreased viability by ~35% (***p < 0.0001) and ~50% (***p < 0.0001) by 15 and 30 μM. Aβ16 demonstrated little impact on cell viability (≤ 5% reduction) at all tested dosages and did not cause substantial toxicity. (B‐D) Fluorescence study using amytracker showed increased fluorescence in Aβ42 treated cells, while hIAPP treated cells showed highest fluorescence and non‐amyloidogenic Aβ16 peptide exhibited the lowest fluorescence levels. Amytracker fluorescence increased modestly in a concentration‐dependent manner following exogenous synthetic Aβ42 and hIAPP exposure but not in Aβ16 treatment. Collectively, these findings validate the differential binding and activity profiles of Aβ42, hIAPP, and Aβ16 in SH‐SY5Y cells. Quantified data in line graphs are reported as mean ± SD from *n* = 4 different experiments.

To evaluate the effects of amyloidogenic peptides on neuronal viability and aggregation behavior, wild‐type SH‐SY5Y neuroblastoma cells were treated with Aβ16, Aβ42, and hIAPP (Figure 5A). Treatment with Aβ42 and hIAPP reduced cell viability, while Aβ16 had minimal effects. Two‐way ANOVA showed significant effects of peptide type (*F* (2,36) = 122.0, *p* < 0.0001) and concentration (*F* (3,36) = 205.8, *p* < 0.0001), as well as a significant peptide‐concentration interaction (*F* (6,36) = 48.06, *p* < 0.0001). Dunnett's multiple comparisons test revealed that Aβ42 reduced cell viability by ~9%, ~20% (***p < 0.0001), and ~65% (***p < 0.0001) at concentrations of 5, 15, and 30 μM, respectively. In contrast, hIAPP produced a modest reduction at 5 μM (~5%, not significant), but decreased viability by ~35% (***p < 0.0001) and ~50% (***p < 0.0001) at 15 and 30 μM. At all concentrations, Aβ16 had minimal effects on viability (≤ 5% reduction) and no significant toxicity was observed. Data were normalized to untreated controls and reported as percentage viability.

To further investigate amyloid aggregation in these cells, we used the Amytracker fluorescence assay in peptide‐treated cultures (Figure 5B–D). Amytracker fluorescence increased in cells treated with Aβ42 and hIAPP, with hIAPP producing the strongest signal, indicating its higher aggregation propensity than Aβ42 (Bharadwaj et al. 2020). Aβ16‐treated cells showed minimal fluorescence at all concentrations, indicating minimal aggregation (Figure 5B–D). Amytracker fluorescence increased with increasing concentrations of Aβ42 (Figure 5B) and hIAPP (Figure 5C), while Aβ16‐treated cells showed comparable fluorescence to untreated controls (Figure 5D). At the highest concentration tested (30 μM), Aβ42 and hIAPP produced fluorescence signals of approximately 12 000 and 33 000 units, respectively, compared to ~3700 units for Aβ16‐treated cells (Figure 5B–D). These results show that Amytracker fluorescence preferentially detects aggregation‐prone amyloidogenic peptides in this cellular system.

These findings support the specificity of Amytracker dye for detecting aggregated, β‐sheet‐rich conformers in live cells and its applicability across multiple amyloidogenic proteins, supporting the broader applicability of this assay to other disease‐relevant amyloids. Together, these results validate the use of Amytracker dye not only for tracking endogenous Aβ aggregation and clearance but also for comparative analysis of diverse amyloidogenic peptides.

## 4 Discussion

Protein aggregation is commonly studied using biophysical methods such as dynamic light scattering (DLS), size‐exclusion chromatography (SEC), and analytical ultracentrifugation (AUC). While powerful, these approaches require purified proteins and are non‐cell based (Chaudhuri et al. 2014). In contrast, imaging‐based aggregation analysis in cells enables visualization of amyloid structures within a biological context (Roberti et al. 2007). Fluorescence‐based imaging approaches often rely on recombinantly produced amyloid proteins as a fusion to fluorescent proteins, but these can potentially alter the aggregation and other functional properties of the amyloid protein. Amyloid‐reactive dyes such as Congo Red and Thioflavin T have been employed to identify fibrillar β‐sheet structures. However, their toxicity, spectral characteristics, and binding specificity are limited, particularly in cellular assays, owing to spectral interference and aggregation inhibition (Álvarez‐Berbel et al. 2025). Several next‐generation probes have been developed to enhance sensitivity and utility in cell and tissue sections. These include X‐34, a derivative of Congo Red (Styren et al. 2000), [[5′‐(4‐Hydroxyphenyl)[2,2′‐bithiophen]‐5‐yl]methylene] ‐propanedinitrile (NIAD‐4), a neutral benzothiazole derivative (Owyong et al. 2025) BF‐188, a benzofuran‐based probe (Ono et al. 2011), curcumin, a natural compound that binds to amyloid fibrils (Velander et al. 2017), and 2‐(1‐ethylidene)malononitrile (FDDNP), a synthetic ligand (Shin et al. 2011). Congo Red, Thioflavin T, and their derivatives (X‐34, NIAD‐4, BF‐188, curcumin, FDDNP) have significantly advanced amyloid detection; however, each possesses notable limitations with specificity, spectral characteristics, cytotoxicity, lack of utility for high‐throughput screening and live monitoring of Aβ accumulation and clearance.

Developing effective drugs for AD has remained a major unmet challenge due to the biological complexity of the disease, slow progression, heterogeneous pathology, and incomplete understanding of its underlying mechanisms, which makes it difficult to identify effective targets and demonstrate meaningful clinical benefit. According to Cummings and colleagues' 2025 Alzheimer's Disease Drug Development Pipeline, 138 distinct compounds are now being investigated in 182 active studies. One‐third of these candidates (~46) are repurposed pharmaceuticals, highlighting the challenges of de novo therapeutic development and the need for more high‐throughput, human‐relevant cell assays targeting Aβ aggregation, toxicity, and clearance (Cummings et al. 2025). Building on our previously established Aβ clearance assay in the MC65 model (Mputhia et al. 2019), this study reports a live‐cell, high‐throughput Amytracker assay capable of monitoring Aβ pathology in cells in real time. LCO dyes such as Amytracker have been shown to selectively bind β‐sheet structures and distinguish aggregate conformers (Urbanek et al. 2025; Pretorius et al. 2018), highlighting both the suitability of Amytracker for detecting amyloid structures and the critical need for the physiologically relevant, live‐cell aggregation and clearance framework established in our study. However, this assay is limited by its reliance on APP‐C99 overexpression and on intracellular Aβ aggregates that may not fully capture extracellular plaque‐like species or the full heterogeneity of Aβ conformers. MC65 cells undergo rapid cell death upon Aβ induction, limiting very long‐term measurements and modeling of slow, progressive AD pathology and not fully recapitulating the metabolic, synaptic, inflammatory, or age‐related complexity of human AD. However, the MC65 Amytracker assay offers a key advantage over existing Aβ cell models by relying on endogenous Aβ production while enabling live visualization of Aβ aggregates, quantitative measurement of its clearance, integrated toxicity readouts, and compatibility with pharmacological screening (Table 1). In this way, the platform fills a critical translational gap, enabling mechanistic characterization and drug screening that cannot be achieved using synthetic peptide models, secreted Aβ assays, or non‐neuronal high‐throughput systems.

**TABLE 1:** Comparative analysis of Aβ cell models.

Model	Cell type	Aβ clearance	High‐throughput	Live monitoring	Endogenous Aβ	Aβ‐induced toxicity	APP processing	3D model	Human CNS‐derived
Synthetic peptide treatment	2D and 3D cell cultures	No	Yes	Yes	No	Yes	No	Yes	Yes
Yeast Aβ Expression Model	Yeast	Yes	Yes	Yes	Yes	No	No	No	No
APP recombinant expression model	HEK293	Yes	Yes	Yes	Yes	No	Yes	Yes	No
APP recombinant expression model	SH‐SY5Y	Yes	Yes	Yes	Yes	No	Yes	Yes	Yes
Primary Neuronal Cultures recombinant expression	Mouse/Rat neurons	Yes	No	Yes	Yes	No	Yes	Yes	No
Primary Neuronal Cultures synthetic peptide treatment	Mouse/Rat neurons	No	No	Yes	No	Yes	No	Yes	No
MC65‐APP‐C99/Aβ Model (This study)	Human neuroblastoma (MC65)	Yes	Yes	Yes	Yes	Yes	No	Yes	Yes

Recent approvals of monoclonal antibody treatments for AD, such as aducanumab, lecanemab, and donanemab show that Aβ lowering can slow cognitive decline (Zhang et al. 2023; van Dyck et al. 2023; Rashad et al. 2022). In parallel with monoclonal antibody‐based therapies, multiple efforts are underway to therapeutically modulate intracellular proteostasis pathways. Several autophagy‐enhancing agents are already in human clinical trials for AD, mild cognitive impairment (MCI), or age‐related cognitive decline, including rapamycin/rapalogs, metformin‐ a diabetic drug, trehalose‐ a natural disaccharide sugar, spermidine‐ a dietary polyamine and kinase inhibitors such as nilotinib and bosutinib (Pagan et al. 2016) are being evaluated in several preventative and early‐AD trials (Riessland and Orr 2023). Notably, many autophagy‐modulating agents now in trials, such as rapamycin, metformin, spermidine, and trehalose, are repurposed drugs or nutraceuticals, rather than AD‐specific autophagy therapeutics, complicating interpretation of target engagement and dosing (Svensson et al. 2024). In contrast to the emerging autophagy‐targeting pipeline, direct proteasome activators remain in the preclinical or early‐translational stage (Pickering 2024). Searches of clinical trial registries and recent pipeline reviews reveal no registered clinical trials evaluating proteasome‐activating small molecules in AD (Gadhave et al. 2016; Checler et al. 2000). Autophagy and proteasome modulators represent a mechanistically rational strategy for AD but face substantial hurdles across pharmacology, target biology, safety, and translational biomarker development. Given the multifactorial nature of AD and the vulnerability of neuronal proteostasis networks in aging, successful modulators will likely need to be highly selective, CNS‐penetrant, capable of restoring downstream lysosomal/proteasomal functionality, and deployable early in disease progression.

## 5 Limitations

The MC65 Amytracker test offers real‐time monitoring of intracellular Aβ aggregation, but has limits to consider. The technique relies on APP‐C99 overexpression and detects intracellular Aβ aggregates, which may not fully recapitulate extracellular plaque formation or the structural heterogeneity of Aβ species observed in real AD brains. Furthermore, the MC65 model's rapid toxicity limits long‐term monitoring of aggregation dynamics and fails to depict the slow, progressive nature of AD pathogenesis. The assay is also performed in a single neuronal cell line; therefore, it does not account for the metabolic, inflammatory, and synaptic interactions seen in the brain. Amytracker dyes accurately detect β‐sheet structures, although fluorescence‐based assessments may have limits in aggregate specificity and quantitative interpretation. As a result, data from this platform should be supplemented with other cellular and in vivo models.

## Author Contributions

**Fraulein Denise Arigo:** writing – original draft, writing – review and editing, methodology, formal analysis. **Ajish Ariyath:** methodology, formal analysis, investigation, writing – original draft, writing – review and editing. **Prashant Bharadwaj:** conceptualization, methodology, project administration, writing – original draft, investigation. **Ralph Martins:** funding acquisition, supervision, project administration. **W. M. A. D. Binosha Fernando:** funding acquisition, writing – review and editing, project administration. **Anna Fyfe:** writing – original draft, writing – review and editing, methodology.

## Funding

Prashant Bharadwaj received funding from NH and MRC‐ARC dementia research development fellowship (APP1107109) for this study.

## Ethics Statement

All the research conducted in this study is covered under ECU Human Research Ethics Committee (HEC) approval and Project Number 14820. The study was performed in accordance with the National Statement on Ethical Conduct in Human Research, Australia (2018 updated).

## Consent

The authors have nothing to report.

## Conflicts of Interest

The authors declare no conflicts of interest.

## Supporting information


**Data S1:** jnc70473‐sup‐0001‐supinfo.pdf

## Data Availability

All data generated or analyzed during this study are included in this published article and its Supporting Information files.
