# Supplementary material for: A High‐Throughput Assay for Monitoring and Quantifying Amyloid‐β Accumulation and Clearance in Alzheimer's Disease Cell Models
Source: J Neurochem. 2026 May 16;170:e70473. doi: 10.1111/jnc.70473 (PMC13179526; doi:10.1111/jnc.70473)
Supplement: Supplementary file 1 — Data S1: jnc70473‐sup‐0001‐supinfo.pdf [file JNC-170-e70473-s001.pdf]

A High-throughput Assay for Monitoring and Quantifying Amyloid-β Accumulation and Clearance in Alzheimer's Disease cell Models

Ajish Ariyath[1, 4] and Fraulein Denise Arigo[1, 4], Anna Fyfe[1], W.M.A.D. Binosha Fernando[1, 4, 6], Ralph Martins[1, 3, 4, 6], Prashant Bharadwaj [1, 2, 4, 6],

[1] Centre of Excellence for Alzheimer's disease Research and Care, School of Medical and Health Sciences, Sarich and Patricia Neuroscience Research Institute, Edith Cowan University, Western Australia, Australia, 6027

[2] Curtin Medical School, Curtin Health and Innovation Research Institute (CHIRI), Faculty of Health Sciences, Curtin University, Western Australia, Australia, 6107

[3] School of Biomedical Science, Macquarie University, Sydney NSW, Australia

[4] Alzheimer's Research Australia, Nedlands, Western Australia , Australia, 6009

[6] University of Western Australia Medical School, Perth, Western Australia, Australia, 6009

*** Corresponding author: Dr. Prashant Bharadwaj, p.bharadwaj@ecu.edu.au**

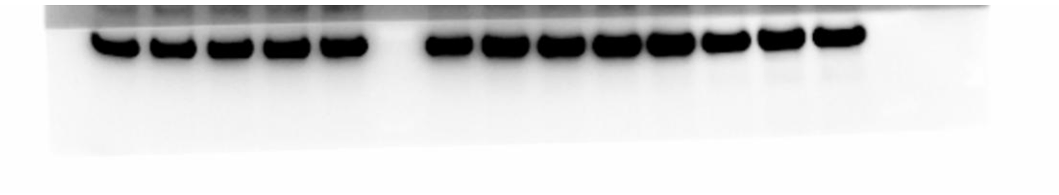

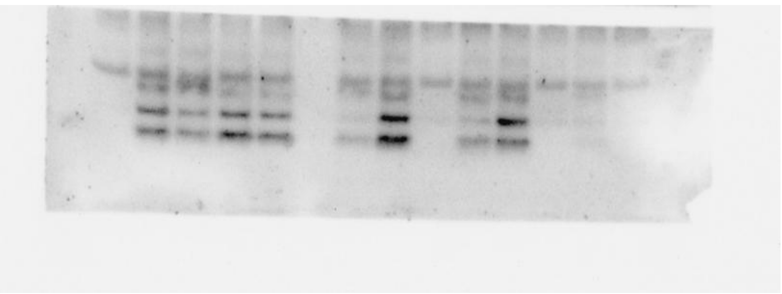

B

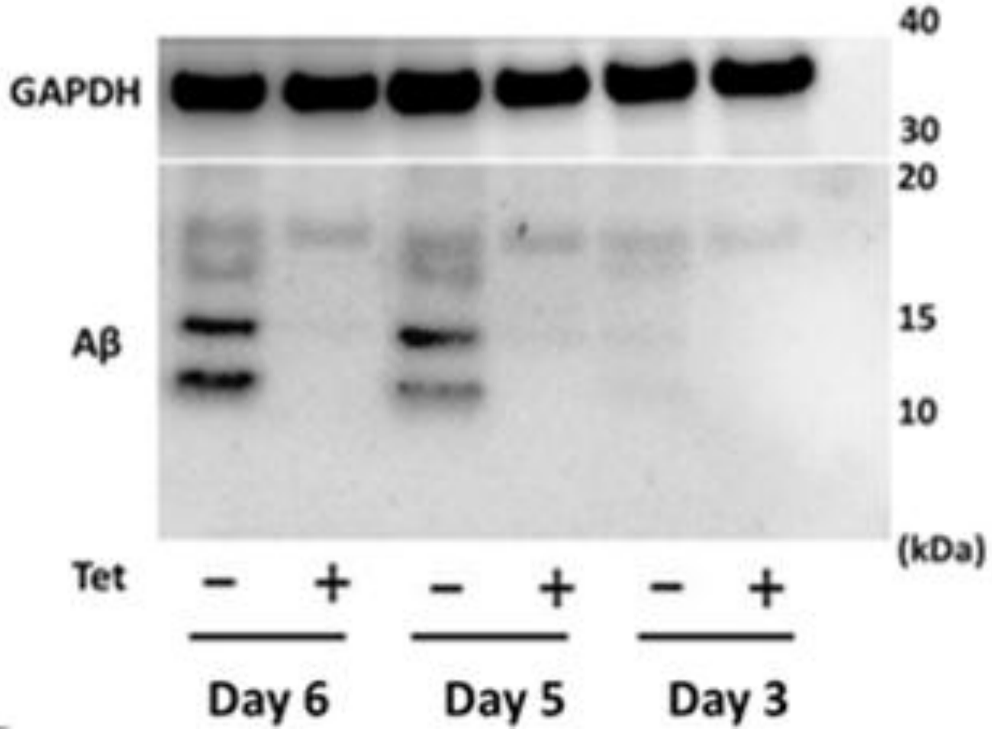

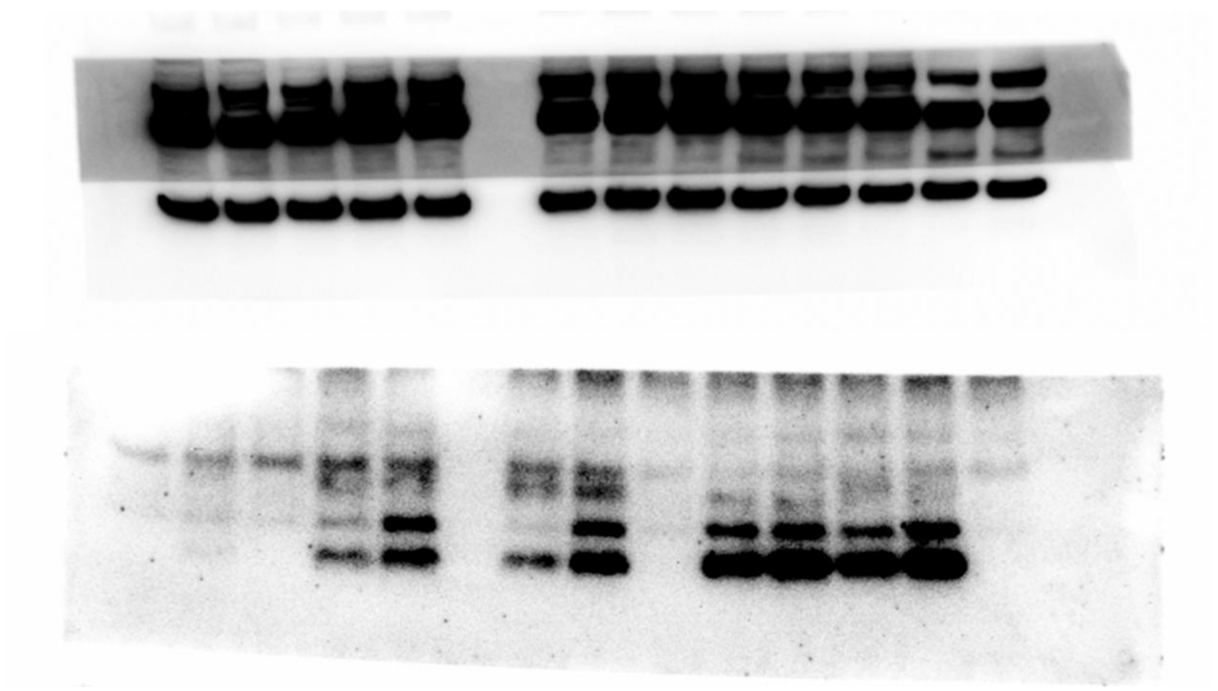

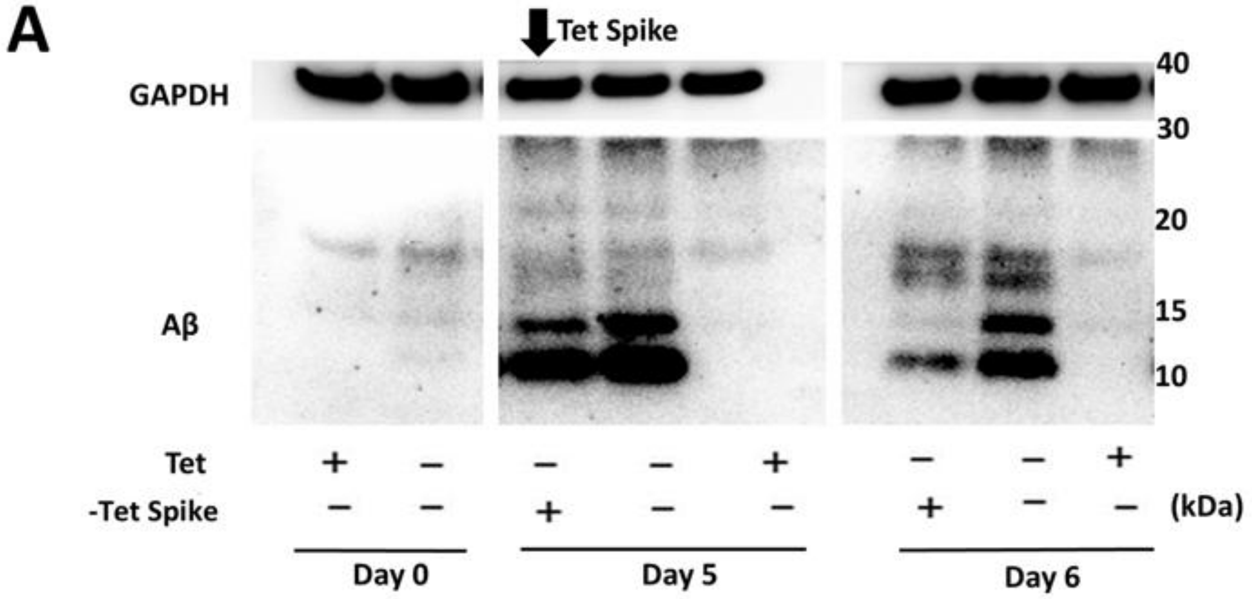
A
Tet Spike
GAPDH
Aβ
40
30
20
15
10
Tet
+ − − − + − − +
-Tet Spike
− − + − − + − −
(kDa)
Day 0
Day 5
Day 6

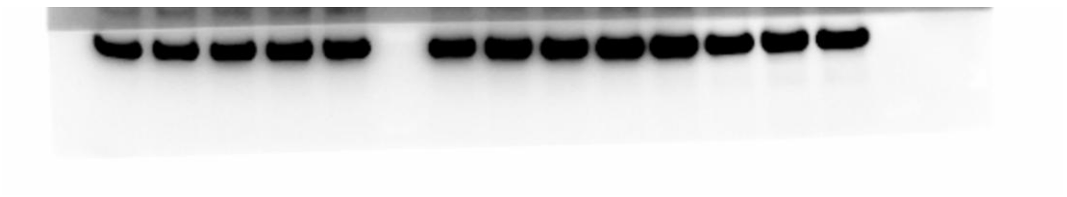

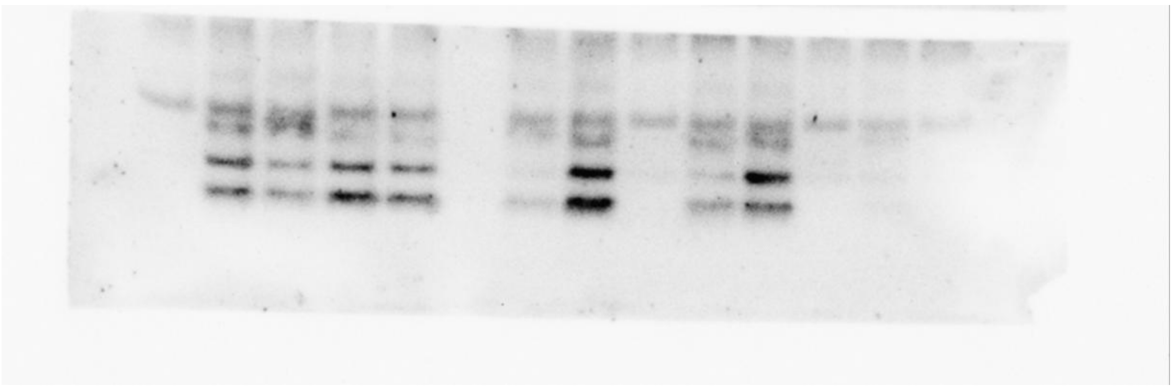

B

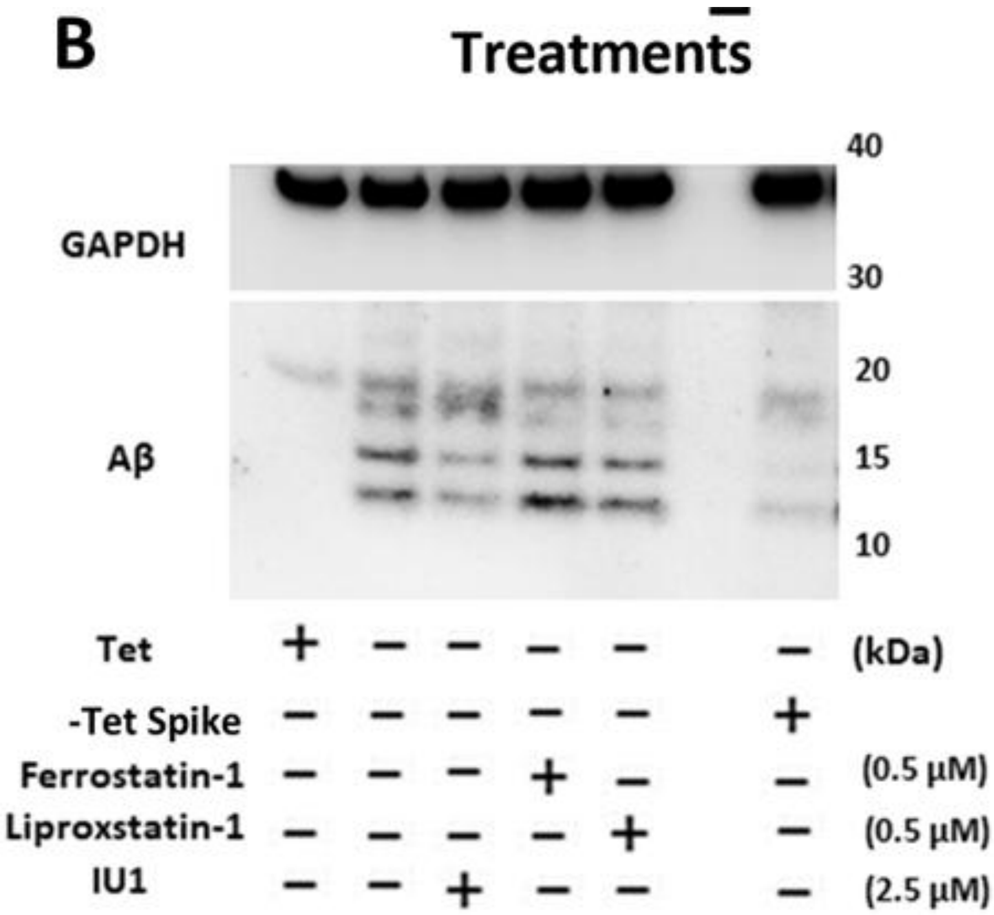
Treatments
GAPDH
Aβ
40
30
20
15
10
(kDa)
Tet + − − − − −
-Tet Spike − − − − − +
Ferrostatin-1 − − − + − − (0.5 μM)
Liproxstatin-1 − − − − + − (0.5 μM)
IU1 − − + − − − (2.5 μM)
